# Autoimmune Polyendocrinopathy-Candidiasis-Ectodermal Dystrophy

**DOI:** 10.3389/fped.2021.723532

**Published:** 2021-11-01

**Authors:** Elise M. N. Ferré, Monica M. Schmitt, Michail S. Lionakis

**Affiliations:** Fungal Pathogenesis Section, Laboratory of Clinical Immunology and Microbiology, National Institute of Allergy and Infectious Diseases (NIAID), National Institutes of Health (NIH), Bethesda, MD, United States

**Keywords:** APECED syndrome, APS-1, AIRE, self-reactive T cells, autoantibodies, type-I interferons, IL-17, IFN-γ

## Abstract

Autoimmune polyendocrinopathy-candidiasis-ectodermal dystrophy (APECED), also known as autoimmune polyglandular syndrome type-1 (APS-1), is a rare monogenic autoimmune disease caused by loss-of-function mutations in the autoimmune regulator (*AIRE*) gene. AIRE deficiency impairs immune tolerance in the thymus and results in the peripheral escape of self-reactive T lymphocytes and the generation of several cytokine- and tissue antigen-targeted autoantibodies. APECED features a classic triad of characteristic clinical manifestations consisting of chronic mucocutaneous candidiasis (CMC), hypoparathyroidism, and primary adrenal insufficiency (Addison's disease). In addition, APECED patients develop several non-endocrine autoimmune manifestations with variable frequencies, whose recognition by pediatricians should facilitate an earlier diagnosis and allow for the prompt implementation of targeted screening, preventive, and therapeutic strategies. This review summarizes our current understanding of the genetic, immunological, clinical, diagnostic, and treatment features of APECED.

## Introduction

APECED is a rare monogenic autoimmune disease (OMIM#, 240300) caused by loss-of-function *AIRE* mutations that impair central immune tolerance and result in the peripheral escape of self-reactive T lymphocytes, which infiltrate various endocrine (e.g., parathyroids, adrenals, gonads, thyroid, pancreas) and non-endocrine (e.g., enamel, stomach, small intestine, lungs, liver, salivary glands, kidneys, spleen, skin) organs and cause autoimmune tissue destruction. Since the discovery of mutations in the *AIRE* gene as the cause of APECED by positional cloning in 1997 (1, 2) and the initial characterization of the immunological functions of AIRE in the thymus in 2002 (3), significant progress has been made in our fundamental understanding of the genetic and immunological basis of AIRE deficiency via the study of APECED patients and of Aire-deficient mice, which develop a multisystem autoimmune disease that closely resembles human APECED particularly on the NOD genetic background, featuring endocrine, non-endocrine, and fungal infection disease manifestations (3–6). In addition, recent patient cohort studies have uncovered an expanded clinical spectrum of APECED (7, 8), which has led to novel observations that may help (a) accelerate diagnosis via earlier recognition of certain disease manifestations and (b) devise effective screening, preventive, and treatment strategies for affected patients. In this review, we present our current knowledge of the genetic and immunological underpinnings of AIRE deficiency and discuss the clinical presentation, diagnostic criteria, and management of APECED patients.

## Genetics of APECED

Bi-allelic loss-of-function mutations in *AIRE* typically underlie APECED with >100 mutations and/or deletions being described throughout the *AIRE* gene (9). Certain APECED cohorts harbor characteristic founder mutations. For example, Finnish, Sardinian, Persian Jew, and Sicilian APECED patients typically carry homozygous p.R257X, p.R139X, p.Y85C, and p.R203X mutations, respectively (9, 10). In the genetically diverse American APECED cohort, compound heterozygous *AIRE* mutations were observed often, with the two most common mutations being p.L323SfsX51 followed by p.R257X (7). In that cohort, p.L323SfsX51, was associated with the development of certain non-endocrine autoimmune manifestations such as pneumonitis and hepatitis (11, 12). Notably, a remarkable variability in the spectrum and severity of the clinical phenotype is seen among APECED patients who carry the same *AIRE* mutations, including among siblings (13–16). This suggests that yet-unknown genetic modifiers may affect individual patient susceptibility to various organ-specific manifestations of the syndrome. To that end, the peripheral tolerance immune checkpoint molecules Cbl-b and Lyn were shown to cooperate with AIRE in modulating the development of autoimmune retinitis and exocrine pancreatitis in mice, respectively (17, 18). In addition, when deficiency in the IL-2/STAT5 response regulatory element CNS0 was combined with AIRE deficiency in mice the result was exacerbation of autoimmune destruction in multiple organs, including in tissues (e.g., adipose tissue) that did not exhibit autoimmunity in isolated CNS0 or AIRE deficiencies (19).

Moreover, several dominant-negative *AIRE* mutations have now been recognized within the SAND and PHD1 domains of the gene and exhibit varying degrees of dominant negative effects *in vitro* and in mice (20–25). Affected patients typically develop milder APECED with fewer autoimmune manifestations compared to patients with classical APECED, while some patients remain unaffected without autoimmunity, indicative of incomplete clinical penetrance (21). Moreover, patients with dominant-negative *AIRE* mutations do not always harbor the cytokine- and tissue antigen-targeted autoantibodies of classical APECED patients (21). Because the minor allelic frequency of some of these mutations (e.g., p.V301M, p.R303Q) is relatively high in the general population, genetic variation in *AIRE* may contribute to the development of organ-specific autoimmune diseases with a greater frequency than previously anticipated. Of interest, a recent genome-wide association study (GWAS) identified two protein-coding AIRE variants (rs74203920; rs2075876) associated with autoimmune Addison's disease, one of which (rs74203920, p.R471C) was also found to be associated with pernicious anemia in an independent GWAS (26, 27).

In recent years, the *cis*-regulatory element CNS1, which promotes AIRE expression in medullary thymic epithelial cells (mTECs) (28, 29) and several regulatory molecules that affect the expression and/or transcriptional activity of AIRE (i.e., HIPK2, FBXO3, JMJD6, SIRT1, DGCR8) have been characterized (30–34). In the subset of patients with a clinical diagnosis of APECED who have wild-type *AIRE* genotype (7), it will be important to examine whether mutations in these or other AIRE regulators and/or partners may underlie their autoimmune disease.

## Pathogenesis of Aire Deficiency

### AIRE-Deficient T Lymphocytes

AIRE is a transcriptional regulator that is highly expressed in a subset of mTECs where it promotes the expression of a large number of -but not all- tissue-specific antigens (3). This process facilitates the negative selection of self-reactive T lymphocytes and the differentiation of self-antigen–specific regulatory T lymphocytes, which collectively shape and maintain central immune tolerance, reviewed elsewhere (13, 14, 35–40). In AIRE deficiency, self-reactive CD4^+^ T lymphocytes escape from the thymus into the periphery and are both necessary and sufficient to cause autoimmune tissue infiltration and destruction, as shown by adoptive transfer experiments of *Aire*^−/−^ CD4^+^ T lymphocytes in immunodeficient mice, by CD4^+^ T lymphocyte depletion experiments in *Aire*^−/−^ mice, and by experiments in *Aire*^−/−^*Tcra*^−/−^ mice (3, 4, 41, 42). The recent discovery and characterization of the post-Aire expressing mTEC subset and of extrathymic Aire-expressing cells (eTACs) in secondary lymphoid tissues require additional functional studies to precisely decipher their contributions in maintaining immune tolerance (43–48). The use of tetramer reagents, epitope mapping, and T cell receptor (TCR) sequencing has helped characterize self-reactive T lymphocyte populations and TCR repertoires in AIRE-deficient mice and/or humans (49–56). As mentioned earlier, AIRE also participates in the positive selection of thymic regulatory T cells (57) and mouse studies have shown that defective neonatal output of thymic regulatory T cells in AIRE deficiency contributes to the development of organ-specific autoimmunity (58). Beyond CD4^+^ T lymphocytes, AIRE-deficient CD8^+^ T lymphocytes and γδ T lymphocytes have also been implicated in the development of certain -but not all- organ-specific autoimmune manifestations in *Aire*^−/−^ mice (i.e., oral candidiasis, pneumonitis, retinitis, peripheral neuropathy) and more work is needed to further define their roles in the breakdown of organ-specific tolerance in cooperation with or independent of *Aire*^−/−^ CD4^+^ T lymphocytes (6, 59, 60).

### Tissue Antigen-Specific Autoantibodies

Furthermore, B lymphocytes are dysregulated in AIRE deficiency associated with expansion of autoreactive naïve B lymphocytes, increased frequency of the CD21^lo^CD38^−^ B lymphocyte subset, and production of a broad array of autoantibodies directed against cytokines and tissue antigens (7, 13, 56). Studies in *Aire*^−/−^ mice have shown that deletion of mature B lymphocytes and their autoantibody-producing potential ameliorates certain organ-specific autoimmune manifestations (41, 42). Whether AIRE-deficient B lymphocytes contribute to autoimmunity via direct priming of AIRE-deficient T lymphocytes and/or via autoantibody production remains unclear (42). Of note, experimental transfer of autoantibody-containing AIRE-deficient serum in mice is not sufficient to promote autoimmunity (41, 42). Serum transfer experiments in the recently developed AIRE-deficient rat model, which harbors a broader spectrum of autoantibodies relative to AIRE-deficient mice, may help further elucidate the potential direct role of autoantibodies in autoimmune tissue destruction (61, 62).

In APECED patients, the detection of several tissue antigen-directed autoantibodies has been associated with the presence of corresponding organ-specific autoimmune manifestations (13, 52, 63–75). New experimental approaches such as phage/bacterial peptide display (PhiP-Seq) and yeast surface display (REAP) have been recently used to uncover novel autoantigen specificities such as KHDC3L, associated with primary ovarian failure, RFX6, associated with intestinal dysfunction, and colipase, associated with exocrine pancreatic insufficiency (76, 77). However, for most of the tissue antigen-targeted autoantibodies detected in APECED patients, it is difficult to reliably predict the development of the corresponding autoimmune manifestation at the individual patient level as their sensitivity and specificity is not very high. Indeed, patients may harbor an autoantibody without featuring the corresponding clinical manifestation while other patients may have a clinical manifestation without harboring the corresponding tissue antigen-directed autoantibody.

Three examples of tissue antigen-directed autoantibodies that can be helpful in the clinical management of APECED patients are worthwhile briefly mentioning. The detection of autoantibodies to 21-hydroxylase in an APECED patient without Addison's disease who did not previously harbor these autoantibodies is often a herald for the forthcoming development of primary adrenal insufficiency (71). Intensified screening with ACTH stimulation testing can help prevent acute adrenal crises in such patients. Moreover, the lung-targeted autoantibodies KCNRG and BPIFB1 have very high specificity (>90%) for autoimmune pneumonitis, although their sensitivity is ~30–60% and, thus, a negative result does not rule out the presence of pneumonitis (11, 52, 74, 78, 79). APECED patients carrying KCNRG- and/or BPIFB1-directed autoantibodies should undergo chest imaging with computed tomography and pulmonary function testing to evaluate for the presence of autoimmune pneumonitis, even if alternative diagnoses (e.g., asthma, bronchitis) were previously made (11). Furthermore, autoantibodies observed in classical autoimmune hepatitis (e.g., anti-LKM, anti-SLA, anti-ASMA) are not typically detected in patients with APECED–associated autoimmune hepatitis (12). Thus, a diagnostic liver biopsy should be performed in APECED patients with persistent transaminase elevation even when classical autoimmune hepatitis-associated biomarkers are negative.

### Autoantibodies Against Type-I Interferons (IFNs)

Beyond tissue antigen-directed autoantibodies, APECED patients harbor neutralizing autoantibodies against certain cytokines, primarily type-I IFNs and type-17 cytokines (80–83). Neutralizing autoantibodies against type-I IFNs, predominantly directed to IFN-ω and the majority of the 13 subtypes of IFN-α, are present in >95% of APECED patients, whereas autoantibodies against IFN-β are detected in ~20% of patients (81, 82), and autoantibodies against IFN-ε are infrequently detected (84). Because IFN-ω-directed autoantibodies are detectable with high sensitivity during infancy before the development of clinical manifestations, and because at that early age these autoantibodies are highly specific for APECED, their early detection carries significant diagnostic utility in children with suspected APECED (81, 82). Of note, IFN-α autoantibodies were proposed to act as ameliorating factors for the development of type-1 diabetes in APECED patients who carry GAD65-directed autoantibodies, indicating that these autoantibodies may also have therapeutic utility (73).

Despite the presence of autoantibodies against type-I IFNs, APECED patients do not develop the severe viral infections that are seen in patients with inherited complete deficiencies of IFNAR1 and IFNAR2 including herpes simplex encephalitis and live attenuated measles-mumps-rubella vaccine-associated disease (85, 86), although ~10–20% of APECED patients have been reported to develop prolonged and/or severe manifestations of cutaneous varicella zoster and/or mucosal herpes simplex infections (87). These clinical observations suggest that APECED patients retain residual compensatory activity of some of the type-I IFNs and/or that alternative type-I IFN-independent immune pathways may provide protection against these viral diseases in the setting of neutralizing autoantibodies against type-I IFNs (88–90). The recent report of severe live attenuated yellow fever 17D vaccine-associated disease in three individuals with neutralizing autoantibodies to type-I IFNs without APECED suggests that this vaccine should be avoided in APECED patients (91).

#### COVID-19 Infection and Vaccination in APECED Patients

Neutralizing autoantibodies against type-I IFNs, particularly to the 13 IFN-α subtypes and IFN-ω, were recently identified in ~10% of patients suffering from critical COVID-19 pneumonia (92) and were shown to delay SARS-CoV-2 clearance (93). This observation was confirmed in independent patient cohorts (94–96). In addition, inborn errors of type-I IFN immunity were reported in some -but not all- examined cohorts of patients with life-threatening COVID-19 (97–99). Given these observations, and the life-threatening pneumonia requiring mechanical ventilation in the first three reported APECED patients with COVID-19 (92, 100), we performed a follow-up international observational study of 22 SARS-CoV-2–infected APECED patients (84). We found that most APECED patients developed severe, hypoxemic COVID-19 pneumonia requiring hospitalization and intensive care unit admission, and four patients (18%) succumbed to the infection (84). These data suggest that the presence of neutralizing autoantibodies to type-I IFNs and the inflammation-prone lung tissue of APECED patients may heighten their risk for life-threatening COVID-19 complications (11, 84, 101). Of interest, another recent study reported four APECED patients who developed mild COVID-19 infection despite the presence of neutralizing autoantibodies to type-I IFNs, consistent with a model of incomplete clinical penetrance (102). These patients were <26-year-old, female, and did not have pre-existing autoimmune pneumonitis (102). Collectively, these reports indicate that APECED patients, particularly adults and/or those with underlying pneumonitis, can be at risk for severe COVID-19. Therefore, APECED patients should be prioritized for vaccination against SARS-CoV-2, which they appear to tolerate without unusual adverse events; however, it is important to note that not all APECED patients develop robust humoral responses to the SARS-CoV-2 vaccine, especially those receiving immunomodulatory therapy (103). In our experience with SARS-CoV-2–infected APECED patients at the NIH Clinical Center, we proceed with prophylactic hospital admission upon diagnosis for close clinical monitoring. In the early ambulatory non-hypoxemic phase of the infection, we consider administration of anti-spike SARS-CoV-2 monoclonal antibodies (104), which was shown to decrease the risk of hospitalization, severe infection, and death from COVID-19 in high-risk individuals without APECED (105). Administration of IFN-β and/or plasmapheresis could also be considered in this setting (84, 106). In the late hypoxemic phase of infection, prompt initiation of corticosteroids is critical to ameliorate lung injury (84, 107), while remdesivir may curtail viral proliferation (108). Caution should be exercised with the use of anti-spike SARS-CoV-2 monoclonal antibodies or IFN-β during the hypoxemic phase of COVID-19, as these modalities could worsen lung inflammation and hypoxemia (109, 110).

### Autoantibodies Against Type-17 Cytokines, IFN-γ-Driven Defects in Oral Epithelial Barrier, and CMC

The presence of neutralizing autoantibodies against type-17 cytokines is associated with CMC, the “signature” infectious disease in APECED patients (80, 83). The majority of APECED patients carry neutralizing autoantibodies against IL-22 (frequency, ~70–90%), whereas neutralizing autoantibodies against IL-17F (frequency ranging from ~20% to ~80% depending on the patient cohort) and IL-17A (frequency, ~35%) are detected less often and neutralizing autoantibodies against IL-17B and IL-17C are not detected (7, 8, 80, 83, 111, 112). However, the association between CMC and the presence of autoantibodies against type-17 cytokines is incompletely penetrant as some patients carry autoantibodies but do not manifest CMC and some others develop CMC without harboring these autoantibodies (7, 8, 80, 83, 113). In fact, in the Russian and American patient cohorts, the frequencies of IL-17 autoantibodies were similar in APECED patients with or without CMC (7, 8), indicating that additional factors must also contribute to CMC susceptibility. Moreover, patients who receive IL-17–targeted monoclonal antibodies (e.g., for psoriasis or inflammatory bowel disease) infrequently develop mild, treatment-responsive oropharyngeal candidiasis (OPC; mean frequency, <10%), as opposed to the ~80–90% frequency of CMC in APECED patients (114, 115). These clinical observations are consistent with the incomplete blockade of IL-17 receptor signaling conferred by these monoclonal antibodies at the mucocutaneous barrier (116, 117), as opposed to the complete abrogation of IL-17 receptor signaling in patients with inherited complete deficiencies of the IL-17 receptors IL-17RA and IL-17RC and of the IL-17 receptor adaptor ACT1 who develop CMC with complete penetrance (118–120). Furthermore, patients with inherited IL-10RB deficiency whose cells do not respond to IL-22 (nor to IL-10, IL-26, IL-28, and IFNL1) do not develop CMC; a single case of treatment-responsive OPC in the absence of iatrogenic immunosuppression has been reported in IL-10RB–deficient patients (OPC frequency, ~3%) who develop very severe early-onset inflammatory bowel disease requiring treatment with corticosteroids and/or TNF-α inhibitors (121–123). Taken together, although APECED patients harbor neutralizing autoantibodies against type-17 cytokines, their presence is unlikely to be the sole factor that might contribute to CMC in the APECED population (124).

This led us examine potential additional mechanisms of CMC susceptibility in AIRE deficiency. Aire-deficient mice, which infrequently harbor neutralizing autoantibodies against type-17 cytokines, were susceptible to OPC despite mounting intact type-17 mucosal responses, indicating that impaired type-17 immunity is not the primary driver of mucosal fungal susceptibility in the model (6). Instead, *Aire*^−/−^ T lymphocytes were both necessary and sufficient to drive OPC susceptibility (6), in agreement with their previously established necessary and sufficient roles in driving susceptibility to all endocrine and non-endocrine autoimmune manifestations of AIRE deficiency in the model (3, 4, 41). Mechanistically, excessive production of IFN-γ by mucosal *Aire*^−/−^ CD4^+^ and CD8^+^ T lymphocytes impaired the integrity of the oral epithelial barrier and promoted OPC, which were both ameliorated by inhibition of IFN-γ and/or JAK/STAT (6). IFN-γ was similarly toxic to human oral epithelial cells *in vitro* and evaluation of mucosal responses in a large cohort of APECED patients, including with RNA-sequencing of oral mucosal tissue in five adult individuals with a history of CMC, showed clear corroborative evidence of exaggerated type-1 responses, while type-17 mucosal responses were intact (6). These findings are consistent with residual compensatory activity of type-17 cytokines in the oral mucosa of the examined patients. Future work will be required to evaluate type-1 and type-17 mucosal responses in infants with APECED before the development of CMC and at the onset of CMC, and to longitudinally study oral mucosal immune responses in patients during acute and quiescent phases of CMC. These studies will help further define the relative contributions of excessive IFN-γ vs. type-17 impairment to the initiation, severity and/or relapse frequency of CMC in APECED patients; it is conceivable that the two mechanisms converge in a subset of APECED patients at different times. Moreover, a clinical trial is being deployed at the NIH Clinical Center to evaluate the safety and efficacy of JAK/STAT inhibition in the management of CMC in APECED patients. Of note, although CMC is the “signature” infection of APECED, these patients are not at risk for invasive candidiasis or other invasive fungal infections, which rely on myeloid phagocytes for effective host defense (125).

Taken together, these findings show that, in certain defined settings, mucosal fungal susceptibility may be driven by aberrant T lymphocyte-mediated immunopathology, not only by impaired type-17 immunity, and support a novel conceptual framework for classifying distinct molecular subtypes of CMC based on the balance between impaired type-17 immunity and/or immunopathology-promoting excessive type-1 inflammation (124).

## Clinical Presentation and Diagnosis of APECED

APECED is clinically defined by the classic triad manifestations of CMC, hypoparathyroidism, and adrenal insufficiency. Developing any dyad among these classic triad manifestations establishes a clinical diagnosis of APECED. Developing a single classic triad manifestation in a patient whose sibling has APECED also establishes a clinical diagnosis. The development of a classic diagnostic dyad raises suspicion for APECED, which then leads to sequencing of the *AIRE* gene and/or testing for autoantibodies against IFN-ω. Several APECED cohorts have been described worldwide with varying disease prevalence. The highest prevalence has been reported in Finnish, Sardinian, and Persian Jew populations (~1:9,000–1:25,000), whereas in the United States, the prevalence of APECED is estimated between 1:100,000–1:300,000 (126).

**Figure 1:**
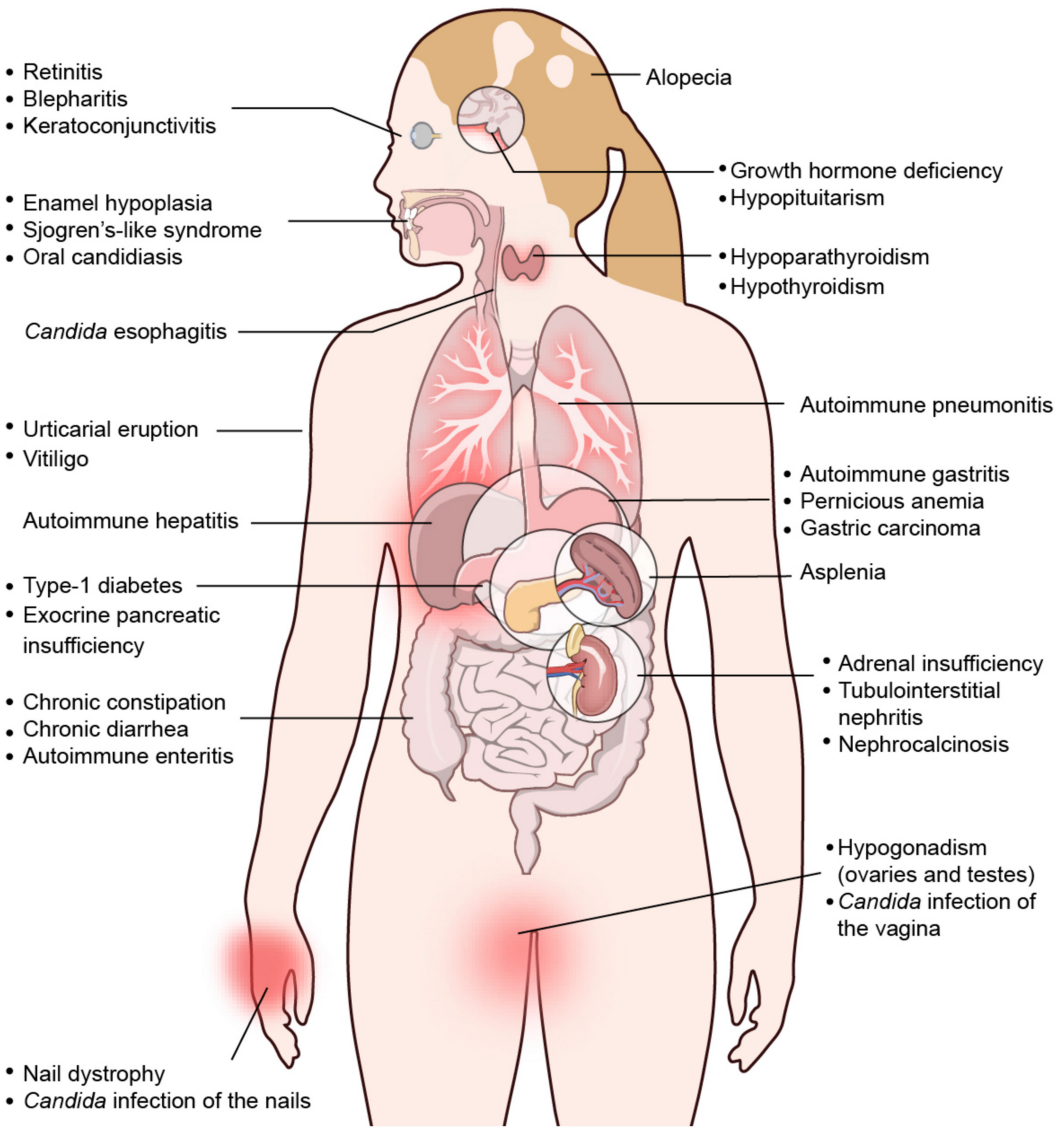
Spectrum of clinical manifestations in APECED patients. Depiction of organ-specific autoimmune manifestations observed with variable frequencies in patients with APECED. Derived from Constantine and Lionakis (13) with permission from Wiley.

**Figure 2:**
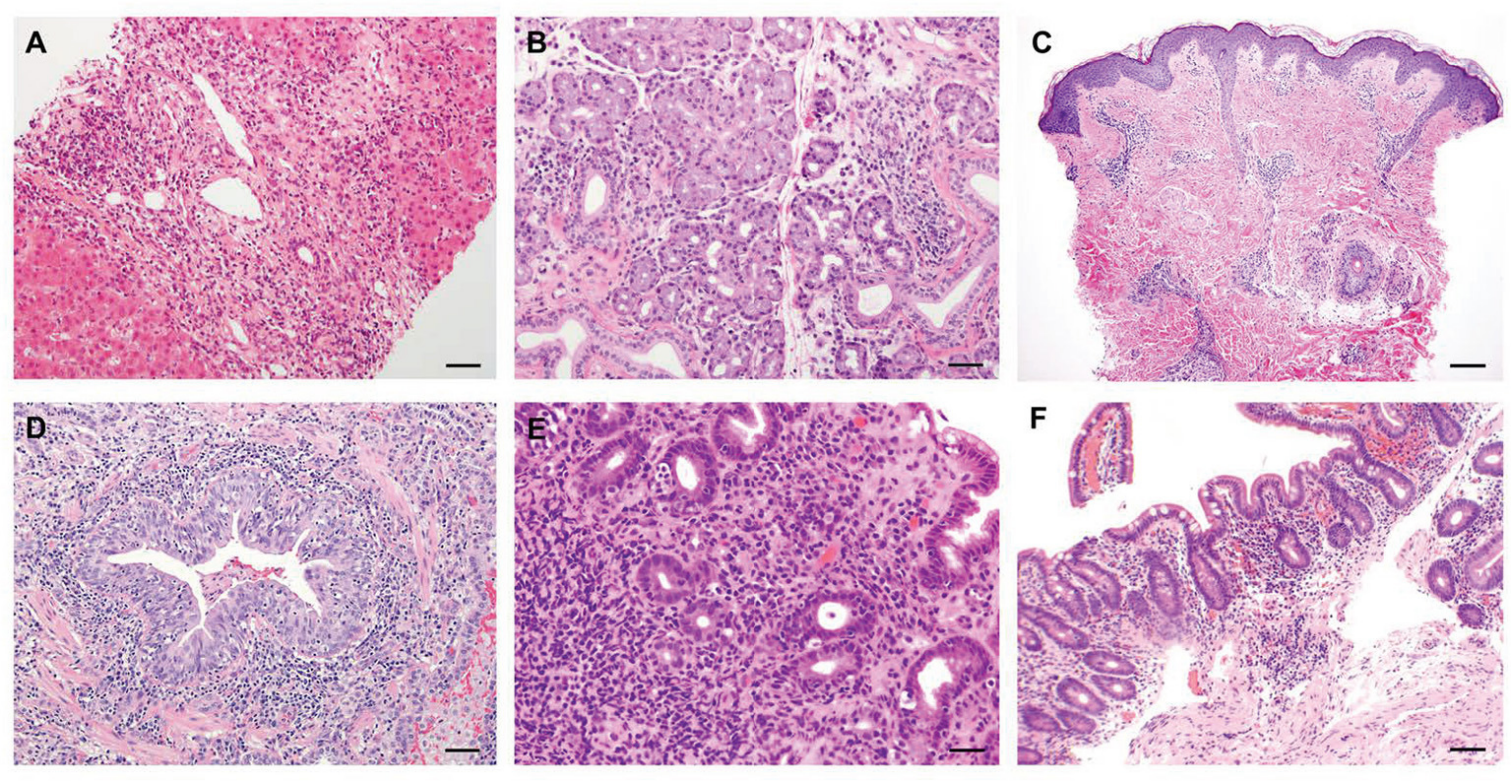
Representative histological features of common non-endocrine manifestations in APECED patients. **(A)** A liver biopsy displaying severe chronic hepatitis with expansion of portal areas by inflammation and fibrosis and extensive interface hepatitis with numerous plasma cells infiltrating into the hepatic parenchyma (H&E; scale bar: 50 μm; original magnification, ×200). **(B)** A minor salivary gland biopsy depicting lymphocytic and plasma cell infiltration in and around the ducts of the gland (H&E; scale bar: 50 μm; original magnification, ×200). **(C)** Skin biopsy of a patient with APECED rash demonstrates perivascular and periadnexal inflammation in the superficial and deep dermis with pallor of the papillary dermis (H&E; scale bar: 2 mm; original magnification, ×40) **(D)** An open lung biopsy revealing chronic bronchiolitis with lymphocytic infiltration within and around the bronchiolar mucosa and lymphoid aggregates in the interstitium nearby (H&E; scale bar: 50 μm; original magnification, ×200). **(E)** A stomach biopsy exhibiting chronic antral inflammation with lymphoplasmacytic infiltrates in the lamina propria and occasionally on glands (H&E; scale bar: 50 μm; original magnification, ×200). **(F)** A jejunal biopsy depicting mild villus blunting and focal acute inflammation (H&E; scale bar: 50 μm; original magnification, ×200). Images in panels A-E are derived from Ferré et al., (7).

**Figure 3:**
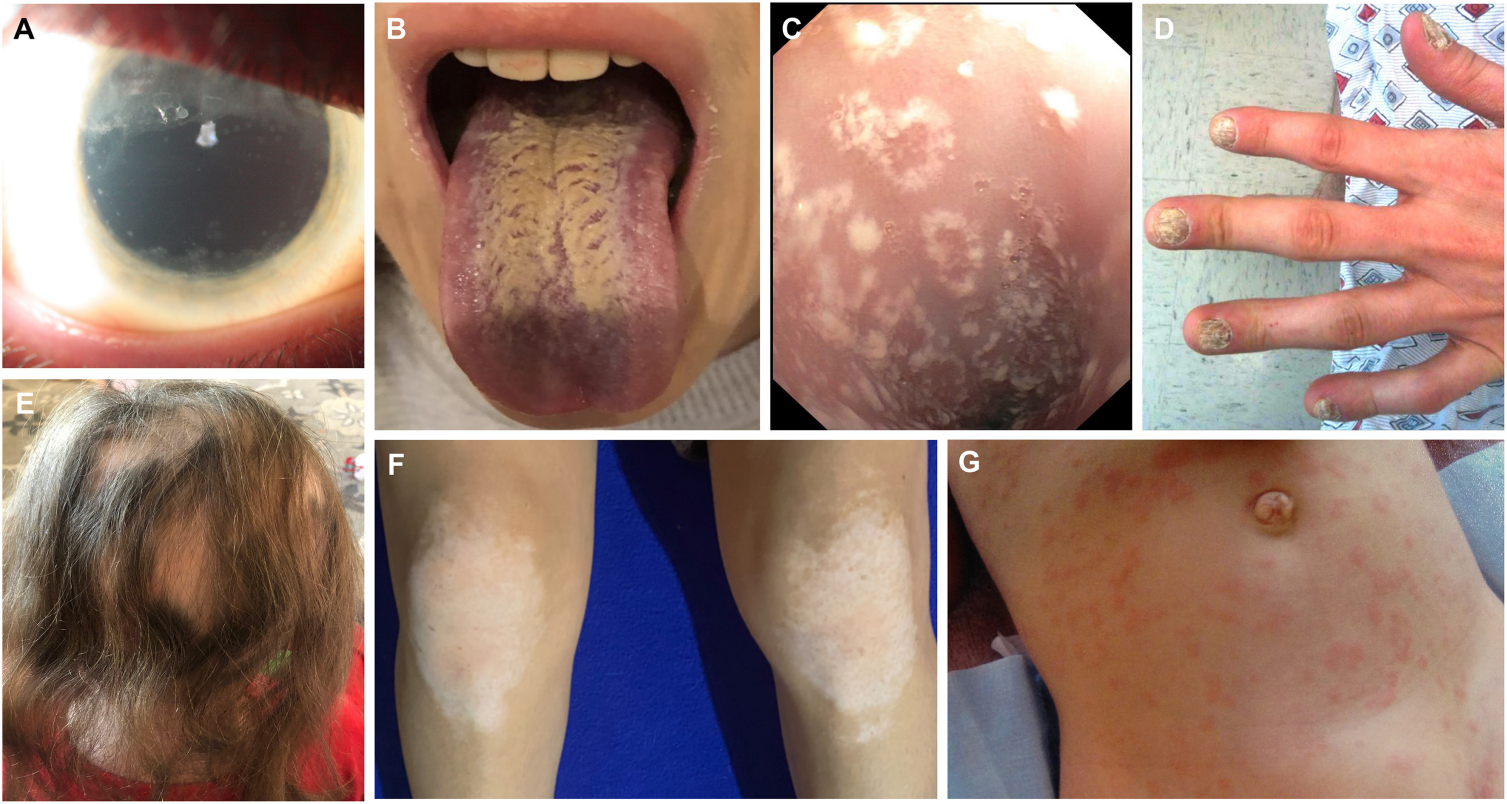
Clinical images depicting ophthalmologic, mucocutaneous and dermatologic manifestations in APECED patients. **(A)** Extensive keratopathy secondary to chronic keratoconjunctivitis. **(B)** Depiction of *Candida* chelitis and thrush affecting the tongue. **(C)** Endoscopic visualization of the esophagus demonstrating *Candida* esophagitis. **(D)** Nail dystrophy. **(E)** Alopecia areata. **(F)** Vitiligo affecting the knees. **(G)** APECED rash in an infant with APECED. Image 3G is derived from Ferré et al., (7).

APECED is a multisystem autoimmune disease that involves several endocrine and non-endocrine organs. More than 30 different autoimmune manifestations have been reported over the past decades with variable, cohort-specific representation of some of these disease components (Figures 1–3); among these manifestations, over 25 involve non-endocrine tissues (7, 8, 13, 112, 126–140). In a prospective natural history study of APECED at the NIH Clinical Center, where we have thus far enrolled >150 patients and evaluated them in a uniform, systematic manner with a multidisciplinary team of clinicians regardless of their underlying clinical manifestations, we have observed a dramatic enrichment of certain non-endocrine autoimmune manifestations relative to other APECED cohorts (7). Specifically, American APECED patients develop a hexad of non-endocrine disease manifestations consisting of urticarial eruption (“APECED rash”), autoimmune gastritis, intestinal malabsorption, autoimmune pneumonitis, autoimmune hepatitis, and Sjögren's-like syndrome with much greater frequency (~40–80%) compared to previously reported European APECED cohorts (<5–20%) (7) (Figures 2, 3). In the American and Russian APECED cohorts that collectively follow >250 patients, several uncommon disease manifestations have also been described, which had not been apparent in previously reported smaller cohorts (8, 141–144).

In the American APECED cohort, we found the mean age of reaching a classic diagnostic dyad to be ~7.5 years; this delay in developing a classic diagnostic dyad is consistent with prior reports (145). Notably, only ~20% of the patients developed their first two consecutive manifestations among the classic triad manifestations. In contrast, the remaining ~80% of the patients developed a median of three non-triad manifestations before eventually reaching a classic diagnostic dyad, therefore resulting in significant delays in establishing a clinical diagnosis (7). Among the non-triad manifestations that occurred early before the development of a classic diagnostic dyad in American APECED patients, three were more prominent: (a) an urticarial eruption (“APECED rash”), which together with CMC were the most common initial disease manifestations, and presented typically as a self-limited, non-pruritic, and often recurrent maculopapular rash with a characteristic histological appearance of combined neutrophilic and lymphocytic dermatosis associated with NLRP3 inflammasome activation but without eosinophilic infiltration or vasculitis (7, 146) (Figure 2); (b) enamel hypoplasia, often featuring early tooth cavity formation, which underscores the importance of close cooperation between dental and medical professionals in the management of APECED patients (147–149); and (c) intestinal malabsorption, which is associated with increased fecal fat, loss of small intestinal enteroendocrine and/or Paneth cells, and gut dysbiosis (150–152). Less frequently, keratoconjunctivitis, autoimmune hepatitis, and autoimmune pneumonitis were observed early in the course of the disease before the development of a classic diagnostic dyad (7, 11, 12) (Figures 2, 3).

These findings led us to propose expanded diagnostic criteria that incorporate the adjunct triad manifestations of APECED rash, enamel hypoplasia, and intestinal malabsorption with the classic triad manifestations (7). With these expanded diagnostic criteria, the development of a diagnostic dyad among the classic and adjunct triad manifestations would be reached ~4 years earlier compared to the development of a diagnostic dyad among the classic triad manifestations, thereby decreasing the time to clinical diagnosis by half (7). In that dataset, accelerated APECED diagnosis by applying the expanded diagnostic criteria would have led to the prevention of life-threatening hypocalcemic seizures and/or adrenal crises in about half of the patients. We have now validated the diagnostic utility of the expanded diagnostic criteria in independent patient cohorts that have been evaluated in our prospective natural history study from both the Americas and non-Nordic European countries (Ferré & Schmitt et al., submitted). In addition, independent re-analysis of previously published APECED cohorts from Finland, Turkey, Sardinia, India, and Brazil indicated that the implementation of our proposed expanded diagnostic criteria would have also resulted in accelerated clinical diagnosis and earlier recognition of APECED in those populations (136–138), attesting to the broader applicability and usefulness of these expanded diagnostic criteria. Future prospective evaluation of patients from other countries in a similar uniform, systematic, multidisciplinary manner will help further define the diagnostic utility of the expanded diagnostic criteria in APECED. An earlier recognition of APECED via the expanded diagnostic criteria could have major clinical implications including (a) early screening for life-threatening endocrinopathies (hypoparathyroidism, adrenal insufficiency), (b) early initiation of treatment for life-threatening non-endocrine autoimmune manifestations (hepatitis, pneumonitis), and, possibly, (c) early administration of prophylactic immunomodulation with the goal to prevent the development of autoimmunity in children.

**Figure 4:**
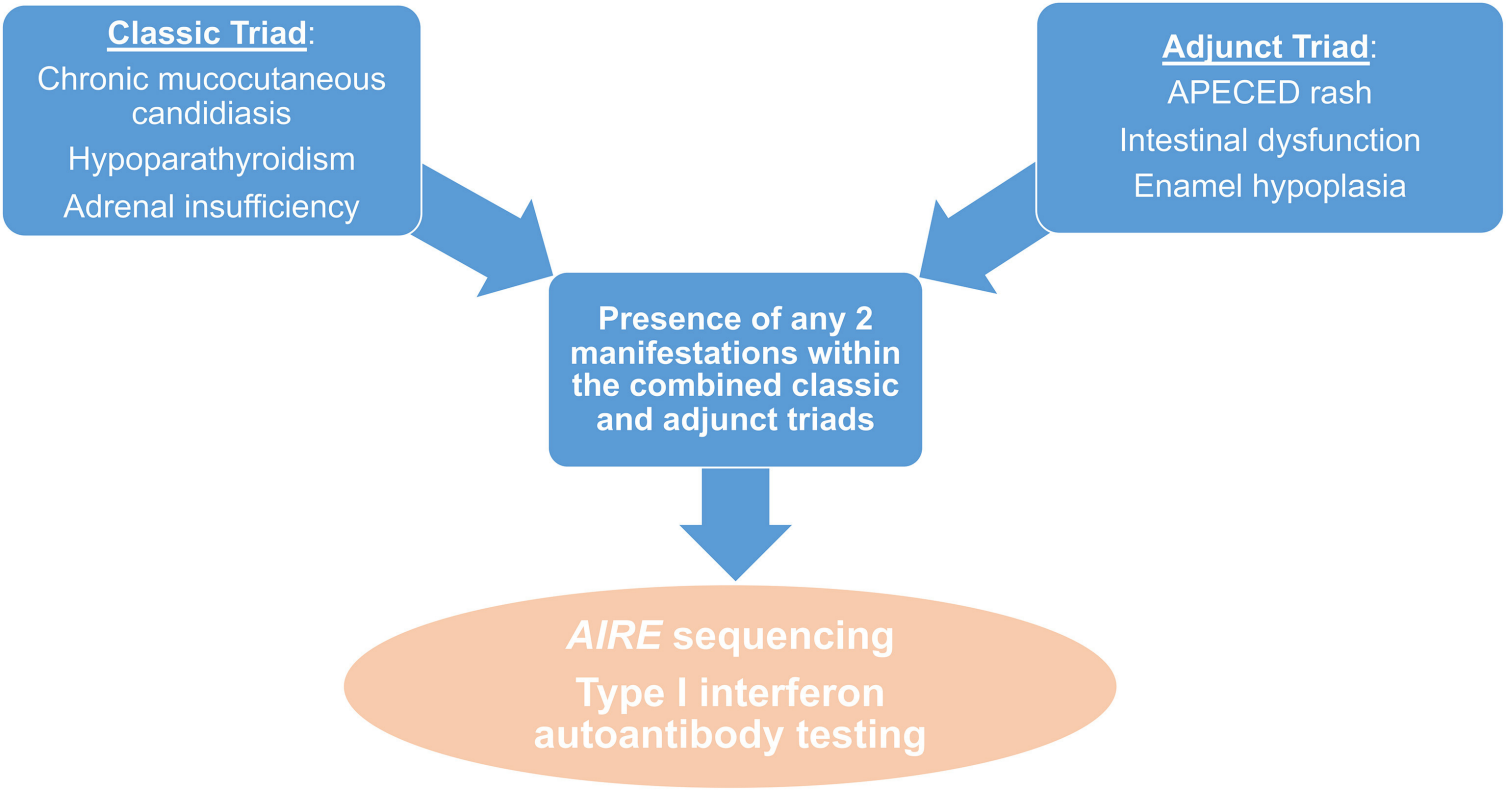
Diagnostic algorithm in patients with suspected APECED. The presence of any 2 manifestations amongst the combined classic and adjunct triads should raise suspicion for APECED and should prompt further work-up with *AIRE* sequencing and measurement of IFNω autoantibodies.

Taken together, these findings show that children affected by APECED in the Americas and other geographic regions are likely to be evaluated by dermatologists, dentists, allergists, immunologists, gastroenterologists as well as hepatologists, pulmonologists, and ophthalmologists early in the course of their disease, not only by endocrinologists who are traditionally more familiar with APECED. The presence of any of the manifestations within the adjunct triad of APECED rash, enamel hypoplasia, and intestinal malabsorption in a child with or without CMC or endocrinopathies or other organ-specific autoimmune manifestations (e.g., hepatitis, pneumonitis, keratoconjunctivitis, other) should raise suspicion for APECED. Such children should undergo a) sequencing of the *AIRE* gene with copy number variation analysis to evaluate for *AIRE* mutations and/or deletions, and b) testing for the presence of autoantibodies against IFN-ω (13, 67, 126) (Figure 4).

## Clinical Management of APECED Patients

The management of APECED patients can be challenging due to the complex medical conditions that they develop, which are associated with poor quality of life and substantial psychosocial burden to both patients and their families (153). Mortality may exceed 30% even with best available medical treatment, driven by adrenal or hypocalcemic crises, end-organ failure (e.g., fulminant autoimmune hepatitis, pneumonitis-associated respiratory failure), malignancies (i.e., oral, esophageal and/or gastric), infections, or suicide (7, 128, 154). Therefore, a coordinated multidisciplinary approach that incorporates several medical and dental specialties is required to provide the best clinical care for the individual patient. Herein, we briefly outline a roadmap of general principles for the management of APECED patients.

### Surveillance for New Manifestations

A critical component of the management of APECED patients is the systematic surveillance for the early detection of new endocrine and non-endocrine manifestations. In patients without hypoparathyroidism, periodic measurement of calcium and intact parathyroid hormone levels can help prevent unsuspected acute hypocalcemic seizures and/or tetany. In patients without Addison's disease, periodic ACTH stimulation testing can help prevent unsuspected acute adrenal crises. Screening for subclinical hypoparathyroidism and/or Addison's disease is particularly important before performing invasive procedures (e.g., esophagogastroduodenoscopy, bronchoscopy) as these can precipitate acute hypocalcemic and adrenal crises in APECED patients with subclinical hypoparathyroidism and Addison's disease. Periodic measurement of thyroid stimulating hormone, glucose, hemoglobin A1c, follicle stimulating hormone, and luteinizing hormone levels can help with early detection of subclinical hypothyroidism, type-1 diabetes, and hypogonadism. Periodic monitoring of transaminases, creatinine, and vitamin B12 levels, chest imaging with computed tomography, and bone density (DEXA) scan can help with early detection of subclinical autoimmune hepatitis, tubulointerstitial nephritis (TIN), pernicious anemia, pneumonitis, and osteopenia, respectively (11, 12, 155). Periodic examination of the oral mucosa by dental specialists can help with early detection of pre-cancerous or cancerous lesions (156). Periodic screening for asplenia, which develops in early adolescence in ~10–20% of patients, includes monitoring for new-onset leukocytosis and thrombocytosis, examination of peripheral blood smears for Howell-Jolly bodies, and/or nuclear liver-spleen scans.

### Management of CMC

Untreated candidiasis can lead to esophageal strictures and may contribute to the development of squamous cell carcinomas of the oral mucosa and/or esophagus; therefore, it is important to treat APECED patients with acute mucosal fungal infection (156, 157). Acute episodes of mucosal candidiasis respond well to induction therapy for four weeks -to reduce the rate of infection relapse following treatment discontinuation- with a triazole such as fluconazole. An echinocandin (e.g., caspofungin, micafungin) is effective when fungal cultures reveal azole-resistant *Candida* strains, which are observed often in patients with APECED and other CMC syndromes (158, 159). In patients with frequent infection relapses (i.e., >3–4/year) in the absence of antifungal prophylaxis, we opt to transition from induction therapy to secondary prophylaxis with swish and swallow amphotericin B solution; in patients with infrequent infection relapses (i.e., <1–2/year), we opt to discontinue antifungal agents at the end of induction therapy and repeat induction therapy when candidiasis recurs. JAK/STAT inhibition has successfully remitted CMC in some patients with STAT1 gain-of-function (160) and it may have a role in the treatment of APECED-associated CMC given the exaggerated mucosal IFN-γ responses that we recently reported in the setting of AIRE deficiency (6); a formal clinical trial has been prepared at the NIH to investigate the efficacy of this treatment modality.

### Management of Endocrine Manifestations

Close monitoring of serum and 24-h urine levels of calcium and phosphorus are needed to optimize the management of hypoparathyroidism. The goals of replacement treatment include the prevention of hypocalcemic crises but also avoidance of the long-term development of nephrocalcinosis, nephrolithiasis, and renal failure resulting from excessive replacement. To that end, maintaining serum calcium levels within the lower range of normal or just below the normal range is favored (161). The recent introduction of recombinant PTH in clinical practice for the treatment of adult patients with hypoparathyroidism (162) may help a subset of APECED patients with hypoparathyroidism to achieve optimal calcium homeostasis, particularly in the setting of pre-existing renal impairment and/or intestinal malabsorption. We recently reported that the peri-procedural use of recombinant PTH in APECED patients was safe and successfully maintained serum calcium levels without the need for intravenous calcium supplementation that can worsen nephrocalcinosis (163). Close monitoring of electrolytes and blood pressure are needed to optimize the management of adrenal insufficiency. The goals of replacement treatment include the prevention of adrenal crises but also avoidance of the long-term development of osteopenia resulting from excessive replacement. All patients with adrenal insufficiency should have access to parenteral hydrocortisone for acute stress dosing and a medical alert bracelet and/or Addison's disease emergency card and should be educated by their treating physicians on the appropriate clinical indications that warrant increasing their hydrocortisone dose (e.g., febrile illness). Furthermore, prompt initiation of hormone replacement is critical to achieve optimal pubertal development and growth in patients with hypogonadism; sperm and oocyte cryopreservation could be considered, when feasible, in adolescent patients before the development of gonadal failure (164).

### Management of Non-endocrine Manifestations

Early diagnosis of certain non-endocrine autoimmune manifestations and initiation of immunomodulatory treatment is critical to prevent the development of irreversible end-organ damage. Autoimmune hepatitis develops in up to 40% of APECED patients, with presentations that may range between asymptomatic laboratory abnormalities to life-threatening fulminant failure requiring liver transplantation (12, 165, 166). A liver biopsy should be performed in APECED patients with persistent elevation in transaminases and/or bilirubin levels, even when classical serological biomarkers for autoimmune hepatitis are negative (e.g., anti-LKM, anti-SLA, anti-ASMA). Histological analysis shows lymphoplasmacytic infiltration and varying degrees of, typically mild, fibrosis in those with autoimmune hepatitis or may reveal an alternative diagnosis (e.g., fatty liver disease) (12) (Figure 2). APECED-associated autoimmune hepatitis responds clinically and biochemically to immunomodulatory treatment. Azathioprine-based or 6-mercapropurine–based therapy is most often used in clinical practice similar to classical autoimmune hepatitis in patients without APECED. In patients with mutations in the thiopurine S-methyltransferase (TPMT) gene that alter the metabolism of azathioprine and result in greater drug exposures and higher risk of azathioprine-induced toxicity, other T-lymphocyte–modulating agents such as mycophenolate, sirolimus, cyclosporine, and tacrolimus have been reported to remit liver inflammation (12). We favor the use mycophenolate or sirolimus in APECED patients due to the renal impairment that cyclosporine or tacrolimus may cause. Of note, we have observed a higher frequency of biopsy-proven mycophenolate-induced colitis in this patient population (~40%) relative to that observed in solid organ transplant recipients (<10%), which requires additional observational studies (167).

Autoimmune pneumonitis develops in up to 40% of APECED patients, typically presenting with chronic cough that is often misdiagnosed as asthma or bronchitis. Chronically untreated pneumonitis progresses to cause severe bronchiectatic structural lung disease with development of secondary pulmonary infections with bacteria and non-tuberculous mycobacteria (11, 168). We perform periodic chest imaging with computed tomography in all APECED patients because some patients can be asymptomatic in the early stages of autoimmune pneumonitis. A bronchoscopy should be performed in patients with radiographic evidence of bronchiectasis and/or ground glass or nodular opacities and/or in patients with abnormal pulmonary function testing, even when BPIFB1 and/or KCNRG autoantibodies are negative. Bronchoscopic findings consistent with the diagnosis of autoimmune pneumonitis include (a) the presence of increased numbers of activated neutrophils in the airways and (b) a thickened basal membrane with increased intraepithelial (CD8^+^ T lymphocytes > CD4^+^ T lymphocytes) and submucosal (CD4^+^ T lymphocytes > CD8^+^ T lymphocytes > B lymphocytes) inflammation in endobronchial tissue biopsies (11). Peribronchial lymphocytic inflammation (CD4^+^ T lymphocytes and CD8^+^ T lymphocytes > B lymphocytes) with B lymphocyte aggregates deeper in the lung parenchyma are typically observed when patients undergo transbronchial tissue biopsies (Figure 2). We recently reported that treatment with the combination of azathioprine (or mycophenolate) and rituximab results in remission of pneumonitis with improvement in clinical symptoms and in radiographic and pulmonary function abnormalities (11). Future studies will be aimed at examining whether targeting the IFN-γ/JAK-STAT axis may ameliorate non-endocrine autoimmune manifestations (including pneumonitis) in APECED patients.

TIN is uncommon (<5–10%) and presents with rapidly increasing creatinine levels that can lead to renal failure requiring kidney transplantation, as opposed to the slower progression of renal impairment that is seen in APECED patients with nephrocalcinosis who do not suffer from TIN (13, 169). Thus, a kidney biopsy should be performed in patients with rapidly evolving renal disease. Azathioprine or mycophenolate, when initiated early, may halt the progression of TIN in some -but not all- patients (13, 169). It is important to note that patients require T lymphocyte-directed immunomodulatory treatment following kidney or liver transplantation because TIN or autoimmune hepatitis will recur in the transplanted organs without immunosuppressive treatment (170, 171). Intestinal malabsorption develops in up to 80% of APECED patients and treatment can be challenging. It is important to rule out exocrine pancreatic insufficiency with measurement of fecal pancreatic elastase-1 levels as these patients respond clinically to pancreatic enzyme replacement therapy. Autoimmune enteritis features lymphocytic infiltration (T > B lymphocytes) in the small intestine and may respond clinically to T lymphocyte-directed immunomodulatory treatment, with sirolimus providing better results (unpublished observations) (Figure 2). *Lactobacillus*-based probiotic therapy was recently reported to ameliorate some gastrointestinal symptoms in a subset of APECED patients based on the presence of gut dysbiosis that is seen in intestinal malabsorption (151). Untreated keratoconjunctivitis can cause blindness and topical application of ophthalmic cyclosporine solution is effective. Patients with asplenia require vaccinations and prophylactic antibiotics to prevent the development of life-threatening sepsis by encapsulated bacteria (172). Anemia is a common laboratory abnormality in APECED patients and may be caused by (a) iron deficiency, which is often associated with intestinal malabsorption and may thus require parenteral supplementation, and/or (b) anemia of chronic disease, and/or (c) B12 deficiency, which requires supplementation to prevent the development of irreversible neurological sequelae, and/or, (d) rarely, autoimmune pure red cell aplasia, which is often refractory to T-lymphocyte–modulating therapy (141, 173).

## Conclusions

APECED is a multisystem autoimmune disease caused by AIRE deficiency, which impairs the negative selection of T lymphocytes in the thymus. In recent years, the characterization of genetically diverse APECED patient cohorts and of dominant-negative *AIRE* mutations that cause milder autoimmune manifestations with incomplete clinical penetrance, and basic studies in Aire-deficient mice have shed more light into the genetics, immunology, clinical presentation, diagnosis, and treatment of AIRE deficiency. Moving forward, improved awareness of APECED among clinicians and a better understanding of the pathogenesis of AIRE deficiency should help devise enhanced strategies for earlier diagnosis and for effective preventive and therapeutic interventions, which should collectively improve the clinical outcomes of affected patients.

## Author Contributions

EF, MS, and ML wrote the manuscript. All authors contributed to the article and approved the submitted version.

## Funding

This research was supported by the Division of Intramural Research of the National Institute of Allergy and Infectious Diseases, NIH.

## Conflict of Interest

The authors declare that the research was conducted in the absence of any commercial or financial relationships that could be construed as a potential conflict of interest.

## Publisher's Note

All claims expressed in this article are solely those of the authors and do not necessarily represent those of their affiliated organizations, or those of the publisher, the editors and the reviewers. Any product that may be evaluated in this article, or claim that may be made by its manufacturer, is not guaranteed or endorsed by the publisher.
